# Metachronous Isolated Splenic Metastasis From Cervical Squamous Cell Carcinoma Directly Invading the Stomach: A Case Report

**DOI:** 10.7759/cureus.80304

**Published:** 2025-03-09

**Authors:** Charalampos Theocharopoulos, Gabriela Stanc, Charalampos-Christos Douligeris, Elissaios A Kontis, Nikolaos Kopanakis

**Affiliations:** 1 Department of Surgery, Metaxa Cancer Hospital, Piraeus, GRC; 2 Department of Pathology, Metaxa Cancer Hospital, Piraeus, GRC

**Keywords:** cervical cancer, oligometastatic cervical cancer, splenectomy, splenic metastases, squamous cell carcinoma

## Abstract

The spleen is a very rare location for isolated blood-borne metastasis from squamous cell carcinoma of the uterine cervix (cSCC), thus splenic metastases from cSCC are associated with diagnostic and therapeutic challenges. We present a case of a 47-year-old woman with a history of International Federation of Gynecology and Obstetrics (FIGO) stage IIIc, human papillomavirus (HPV)-associated cSCC who presented with an isolated splenic metastasis eight months after completing primary treatment. The patient presented to the emergency department with symptomatic anemia. A CT scan of the abdomen showed a large splenic mass measuring 6.8 x 6.8 cm that appeared to directly invade the fundus of the stomach; a subsequent gastroscopy revealed an ulcerated, oozing lesion, which was biopsied and confirmed to be SCC. Following a multidisciplinary tumor board discussion, given the inability to obtain endoscopic hemostasis, the patient underwent expedited splenectomy, distal pancreatectomy, longitudinal gastrectomy, and pyloroplasty. Histological examination showed a high-grade, HPV-associated cSCC, consistent with metastatic spread from the known primary cervical cancer. The patient was initiated on cisplatin, paclitaxel, and pembrolizumab and received four cycles before experiencing disease progression.

## Introduction

Cervical cancer (CC) is the fourth most common malignancy in women and the second most common among women of reproductive age worldwide [1]. Despite advances in prevention, screening, and treatment over the last decades, CC remains the second-leading cause of cancer-related death in women aged 20-39 years [2]. Squamous cell carcinoma (SCC) is human papillomavirus (HPV)-associated in more than 95% of cases and accounts for approximately 70% of CC cases [3]. In a large series of 1347 patients with metastatic cSCC, distant metastases were primarily single-site (68.7%), occurring most commonly in the lung (37.9%), bone (16.7%), and liver (12.5%); multi-organ metastases involved both the lung and the liver or the bone [4]. Notably, splenic metastases were not reported in this study. Splenic metastases from epithelial tumors are exceedingly rare, occurring in up to 1% of cancers with metastatic spread [5].

Hereafter, we present a case of a 47-year-old woman with SCC of the uterine cervix who presented with a solitary metachronous splenic metastasis causing significant upper GI bleeding, eight months after index treatment. The patient was managed surgically followed by systemic therapy. Part of this article, excluding pathological findings, histopathology images, and critical literature discussion, was previously presented as a meeting abstract at the 43rd Congress of the European Society of Surgical Oncology on October 2, 2024. We also concisely review the current evidence for the management of metachronous splenic metastasis from cSCC.

## Case presentation

A 47-year-old woman presented to the ED with a three-week history of weakness, tiredness, and shortness of breath, which had progressively worsened over the past week. She was found to be severely anemic, with a hemoglobin level of 5.9 g/dl (reference range: 12-15 g/dl), but was hemodynamically stable. Her relevant past medical history included treatment in another country for a FIGO stage IIIc, HPV-associated cSCC, with combined cisplatin-based chemotherapy and radiotherapy at a dose of 50.4 gray (Gy) in 27 fractions, followed by intracavitary brachytherapy at a dose of 27 Gy in three fractions. As the patient had received treatment in another country, obtaining exact treatment details was challenging. According to the patient, treatment had concluded eight months prior to admission and resulted in complete remission.

**Figure 1:**
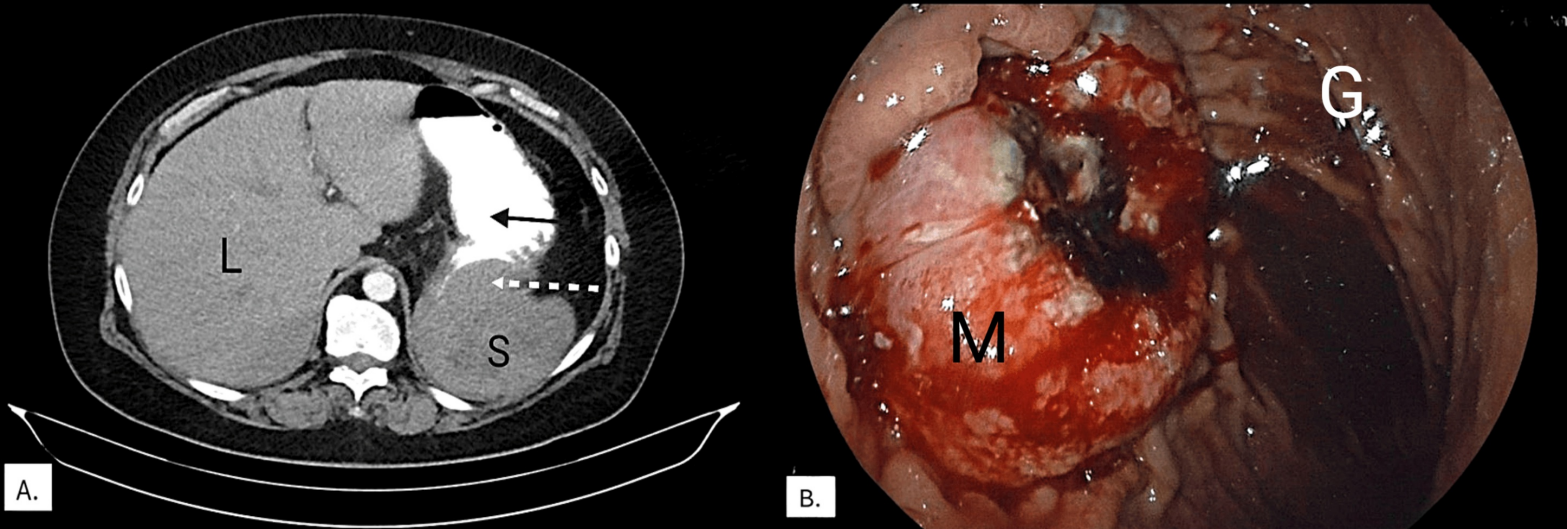
Imaging and endoscopic findings. A. Abdominal CT with IV and PO contrast shows a large splenic mass measuring 6.8 x 6.8 cm invading the gastric fundus. L: liver; S: spleen; Black arrow: stomach after oral contrast ingestion; White dashed arrow: splenic mass infiltrating the gastric fundus. B. Gastroscopy shows a slowly oozing, ulcerated lesion measuring 6 x 6 cm, representing full-thickness wall infiltration. M: splenic metastasis fully invading the gastric wall; G: normal gastric mucosa.

**Figure 2:**
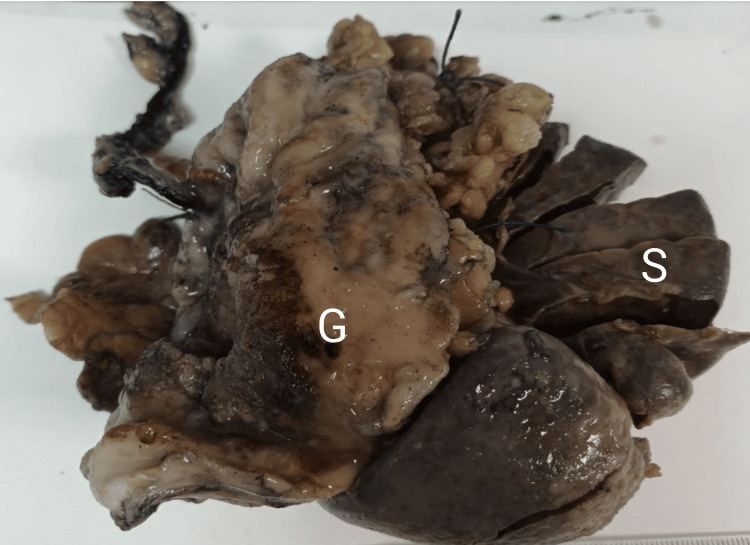
Surgical specimen. En bloc surgical specimen of longitudinal gastrectomy and splenectomy; the pancreas is not visualized. S: spleen; G: stomach.

The initial treatment of the patient included transfusions of two units of packed red blood cells and one unit of fresh frozen plasma. An abdominal CT scan with per os and intravenous contrast administration showed a large splenic mass measuring 6.8 x 6.8 cm directly invading the posterior wall of the gastric fundus (Figure 1A). Stranding surrounding the superior cervix was noted, along with endometrial wall thickening (maximum diameter: 2.5 centimeters) with fluid density, potentially representing local recurrence. Chest CT did not yield evidence of metastases. A subsequent upper GI endoscopy showed an ulcerated, slowly oozing lesion of the fundus measuring 6 x 6 cm (Figure 1B); the obtained biopsies confirmed SCC. The patient was discussed in an ad hoc, same-day multidisciplinary tumor board, which reached a consensus to pursue surgical intervention, with a view to control the hemorrhagic source. Salvage surgery for the potential local recurrence was not deemed appropriate at the time due to the systemic dissemination of the disease, the urgent need for hemorrhage management, and the history of field radiation, which significantly increased the risk of intraoperative and postoperative complications. The patient underwent an expedited laparotomy, where splenectomy, distal pancreatectomy, longitudinal gastrectomy, and pyloroplasty were performed (Figure 2). The total duration of the operation was 240 minutes and the approximate blood loss was 500 milliliters. The patient received three units of packed red blood cells, two units of fresh frozen plasma, and was administered IV norepinephrine due to intraoperative hemodynamic instability. The patient was discharged on the tenth postoperative day. Her postoperative course was complicated by delayed gastric emptying, necessitating the use of a nasogastric tube for five days. She subsequently resumed oral feeding.

**Figure 3:**
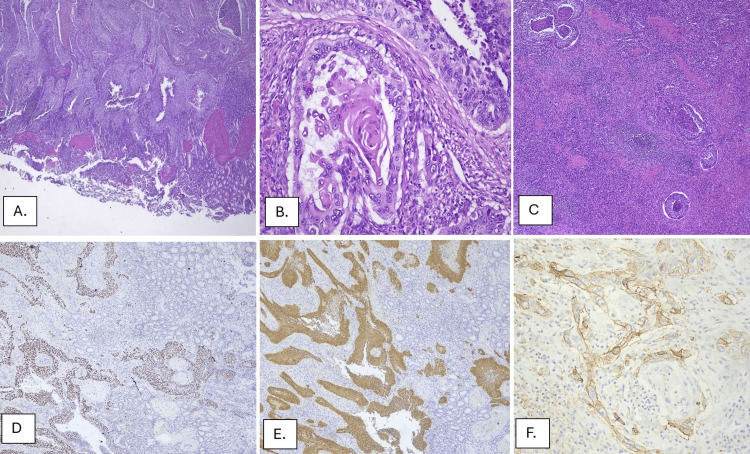
Histopathological findings. A. H&E, x20: Poorly differentiated metastatic squamous cell carcinoma with pronounced nuclear atypia and central necrosis, infiltrating the stomach. B. H&E, x200. C. H&E, x40: SCC invading the spleen parenchyma. D. IHC, x40: Tumor cells positive for p40. E. IHC, x40: Tumor cells positive for p16. F. IHC, x200: Tumor cells positive for PD-L1, CPS > 1 (CPS = 15.5). IHC: Immunohistochemistry; SCC: Squamous Cell Carcinoma; CPS: Combined Positive Score; PD-L1: Programmed Death-Ligand 1.

On gross examination, the surgical specimen consisted of an en bloc resection comprising a longitudinal gastrectomy specimen measuring 12 x 7 x 5.5 cm, the spleen measuring 11 x 7 x 4.5 cm, the tail of the pancreas, and a segment of the greater omentum. On dissection, the tumor was found to arise from the splenic parenchyma, extending contiguously to involve the adipose tissue in the hilum region, infiltrating the full thickness of the stomach wall and ulcerating the mucosa over an area of 5.5 x 4 cm. The tail of the pancreas and the associated lymph nodes had no evidence of metastatic infiltration. Microscopically, the tumor was a viable, invasive, keratinizing SCC, poorly differentiated, HPV-associated, with focal basaloid morphological features (<50%) and extensive comedo necrosis (Figure 3). The surgical margins of the stomach and all resected lymph nodes were free of neoplastic invasion. On immunohistochemical examination (IHC), tumor cells were diffusely positive for CK903 (34βE12), p40, p16, EMA, CK5/6, CK7, and CK8/18 (Cam5.2). Sections examined from the spleen and the stomach showed a similar tumor morphology. Based on the morphology and IHC, a diagnosis of metastatic SCC was made according to the clinical history of a known uterine cervix SCC.

The patient was initiated on cisplatin (75 mg/m^2^), paclitaxel (175 mg/m^2^), and pembrolizumab (200 mg); unfortunately, she experienced disease progression after receiving four cycles, with liver metastases. At this point, given her poor performance status and the natural history of the disease, she was offered the best supportive care and eventually expired eight months after the operation.

## Discussion

This case represents a rare clinical scenario with inherent diagnostic and therapeutic challenges. The differential diagnosis of SCC contiguously involving the spleen and the stomach could also be attributed to a primary SCC of the stomach (gSCC). Differentiating gastric and metastatic cervical SCC should be done using clinical and IHC characteristics. Primary gSCCs are extremely rare entities, accounting for 0.04% to 0.07% of gastric cancers with a male to female ratio of 5:1 [6]. Distinguishing metastatic cSCC from gSCC using IHC relies on identifying markers specific to the cervical origin versus those typically seen in gSCC. P16 is a surrogate marker for HPV infection and is positive in more than 90% of cSCC compared to less than 65% in gSCC [7,8]. Furthermore, the majority of SCCs of various origins are negative for CK7, whereas cSCC is positive in 87% of cases, including our patient [9]. Conversely, CK20 is generally negative in cSCC and occasionally positive in gSCC. Thus, in a female patient with a history of cSCC, this case was considered to be metastatic. The patient was managed on an 'emergency basis' due to clinically significant hemorrhage, which could not be controlled with endoscopic or interventional radiology techniques. Surgical resection was offered to the patient to obtain robust control of the bleeding source.

Current evidence from patient series and case reports on hepatic and pulmonary resections for isolated parenchymal metastases suggests a potential survival benefit [10]. Limited evidence indicates that patients with non-liver metastases may have a better outcome as opposed to those with liver metastasis [11]. According to the 2023 European Society of Gynaecological Oncology (ESGO)/European Society for Radiotherapy & Oncology (ESTRO) guidelines, oligometastatic recurrence can be treated with surgical resection, thermal ablation, brachytherapy, or external radiotherapy, based on the size and site [12]. Metachronous oligometastatic SCC recurrence was associated with better progression-free (PFS) and disease-specific survival (DFS) compared to multi-organ spread (HR: 2.95 (1.23-7.08) and HR: 3.28 (1.40-7.70), respectively) [13]. In cases of oligometastatic recurrence, metastasectomy with or without adjuvant therapy reduced the risk of second recurrence and improved PFS compared to palliative chemotherapy alone (OR: 0.15 (0.02-0.92) and HR: 0.24 (0.06-0.99), respectively) [13].

These outcomes are reflected in the published cases of solitary SCC splenic metastases [14]. All cases of isolated splenic metastases were treated with splenectomy followed by adjuvant chemotherapy except for the case of Goktolga U et al. [15], where splenectomy was not feasible due to invasion of the hepatic artery and vein. Importantly, all six patients who underwent splenectomy and had a follow-up greater than one year achieved a 12-month DFS. Conversely, patients with extra-splenic organ metastases were managed with palliative chemotherapy and had a worse disease course, as two out of three patients died within four months [16-18]. In our case, the patient received four cycles of chemotherapy before experiencing disease progression and expiring eight months after the operation.

Based on these data, in the case of our patient, apart from controlling the bleeding source, surgical resection was the indicated treatment for oncological purposes. Metastasectomy appears to be superior to conventional chemotherapy alone and should therefore be the preferred treatment modality in such cases. Alternative treatment options in cases of isolated splenic metastases include systemic pharmacotherapy, radiofrequency or microwave ablation, and stereotactic body radiotherapy (SBRT). Newer agents, including immune-checkpoint inhibitors (ICIs) and antibody-drug conjugates (ADCs), have produced better outcomes compared to conventional chemotherapy alone in recurrent/metastatic cervical cancer and could offer a survival benefit in this patient population [19-21]. Thermal ablation of splenic metastases with curative intent has been reported in several case series and appears to be a feasible alternative, allowing for splenic conservation [22,23]. However, no cases of ablative procedures for splenic metastasis from cSCC have been reported to date. Similarly, data for SBRT in the treatment of splenic metastases are scarce. Published studies, primarily from non-small cell lung cancer, report promising results with durable local control, warranting further investigation [24]. SBRT for oligometastatic pulmonary lesions from cSCC resulted in a 1-year overall survival (OS), PFS, and local control rates of 76.8%, 55.8%, and 75.6%, respectively [25].

Splenic metastases from epithelial tumors in principle are rare, and the underlying mechanisms contributing to this have not been fully elucidated. Studies in blood cell types, except for red blood cells, have shown a prolonged intrasplenic vascular transit time (mean: 10 minutes), which would be expected to enable circulating tumor cells (CTCs) to successfully extravasate and seed metastases [26]. Follain G et al. demonstrated in vivo that pro-metastatic vascufet lar niches exhibit intermediate blood flow profiles, which are sufficiently low to enable CTC arrest and endothelial adhesion, yet high enough to stimulate endothelial remodeling that facilitates CTC extravasation [27]. Elevated transit times are also observed in the lungs and liver, organs where blood-borne metastasis from carcinomas are common [26]. Most published hypotheses aiming to explain the rarity of splenic metastases appear unconvincing, suggesting that the contraction of the splenic capsule and the acute angle of the splenic artery branching from the celiac trunk act as protective mechanisms [28]. A more recent hypothesis suggests that during this physiologic splenic pooling, cancer cells are exposed to pro-apoptotic signals and are destroyed, similarly to granulocyte apoptosis [26]. Further studies are needed to elucidate the rarity of splenic metastases.

## Conclusions

Overall, splenic metastases from cSCC are rare; however, they should always be included in the differential diagnosis for patients with a known history of malignancy and newly identified splenic lesions. In cases of isolated, resectable metastases, splenectomy followed by adjuvant chemotherapy appears to be an effective option that prolongs patient survival. Further evidence from larger patient series is required to consolidate the role of surgery in these patients, either as a modality that offers oncologic benefit or as an emergency measure in acutely symptomatic cases. Multi-organ metastatic disease carries a poorer prognosis and should be managed with chemotherapy alone.
